# The Effect of Temperature on Shear Bond Strength of Clearfil SE Bond and Adper Single Bond Adhesive Systems to Dentin

**Published:** 2015-03

**Authors:** Farahnaz Sharafeddin, Hossein Nouri, Fatemeh Koohpeima

**Affiliations:** a Dept. of Operative Dentistry, Biomaterials Research Center, School of Dentistry, Shiraz University of Medical Sciences, Shiraz, Iran.; b Undergraduate student, School of Dentistry, Shiraz University of Medical Sciences, Shiraz, Iran.; c Dept. of Operative Dentistry, School of Dentistry, Shiraz University of Medical Sciences, Shiraz, Iran.

**Keywords:** Adhesive, Temperature, Shear Bond Strength

## Abstract

**Statement of the Problem:**

Monomer viscosity and solvent evaporation can be affected by the adhesive system temperature. Higher temperature can elevate the vapor pressure in solution and penetration of adhesive in smear layer. Bonding mechanism may be influenced by the adhesive temperature.

**Purpose:**

This study aimed to evaluate the effect of pre-heating on shear bond strength of etch-and-rinse and self-etching adhesives to ground bovine dentin surfaces, at temperatures of 4˚C, 25˚C and 40˚C.

**Materials and Method:**

In this experimental study, 60 maxillary bovine incisors were randomly divided into 6 groups (n=10). The central part of labial dentin surfaces was exposed with a diamond bur and standardized smear layer was created by using silicon carbide paper (600 grit) under water-coolant while the specimens were mounted in acrylic resin. Two adhesive systems, an etch-and-rinse (Adper single bond) and a self-etch (Clearfil SE Bond) were stored at temperatures of 4˚C, 25˚C and 40˚C for 30 minutes and were then applied on the prepared labial surface according to the manufacturer’s instructions. The composite resin (Z350) was packed in Teflon mold (5 mm in diameter) on this surface and was cured. The shear bond strength (MPa) was evaluated by universal testing machine (Zwick/Roell Z020, Germany) at cross head speed of 1mm/min. The results were statistically analyzed by using ANOVA and Tukey tests (*p*< 0.05).

**Results:**

No significant difference was found between the shear bond strength of Clearfil SE Bond adhesive in different temperature and single Bond adhesive system at 25 ˚C and 40 ˚C. However, there were significant differences between 4 ˚C of Adper single bond in comparison with 25˚C and 40˚C (*p*= 0.0001).

**Conclusion:**

Pre-heating did not affect the shear bond strength of SE Bond, but could promote the shear bond strength of Adper Single Bond.

## Introduction

Intimate contact is the requisite for optimum adhesion in presence of liquid adhesive and solid adherent. [1-3] The adhesive agent acts as an intermediate layer to adhere the resin restorative to the dentin surface through a chemical reaction or leading a micro-mechanical retention of the dentin surface. [4] Raising the temperature may promote the radical activity and polymerization process and would lead to producing a polymer with a greater cross-linking network and higher degree of conversion. [5-7]

Cold adhesive in refrigerator or warm adhesive in warmer area stimulates chemical reaction and affects the quality of bonding to enamel and dentin and its stability. [8]

The chemical contents of adhesive systems in different temperature must be considered [9] because heating enhances some characteristics including viscosity and degree of conversion which are the two main factors that affect the bonding quality. [10] For example in acetone-based adhesives, the degree of conversion enhances at higher temperature. [11] One of the important particularities of adhesive systems is its low viscosity due to the presence of their solvents and diluted monomers, which enables them to penetrate deeper. [12]

As a result, low temperatures can decrease the evaporation of the solvent and water. Low bond may lead to the presence of the residual solvent and water in the bonding layer. [10, 13-14] Bonding mechanism can be influenced by self-etching adhesive. Lower temperature can cause disintegration of the smear layer and dentin demineralization by reducing the adhesive viscosity. [9]

It was reported that pre-heated self-etching adhesives would reduce the bond strength. It has also been reported that higher temperature can elevate the vapor pressure in solution and enhance the solvent evaporation. This evaporation decreases the contraction of water in the complex, and would reduce the demineralization ability of the adhesives by disintegrating the smear layer and demineralizing the products. [10]

The effect of the temperature of adhesive agent on shear bond strength has rarely been investigated. Hence, the aim of this study was to evaluate the effect of preheated self-etch and etch-and-rinse adhesives on the shear bond strength of composite restorations to dentin. 

## Materials and Method

This experimental study assessed the self-etching adhesive Clearfil SE Bond (Kureray medical Inc; Kurashiki, Japan) and etch-and-rinse adhesive system Adper Single Bond (3M ESPE; St. Paul, MN, USA). 

Sixty extracted, noncarious bovine incisors were cleaned and stored in 0.1% thymol solution for one week. The roots and the enamel of central part of labial surface were removed and the labial dentin surface was exposed by using a diamond bur (4138, KG Sorensen; Barueri, SP, Brazil) and a high-speed handpiece under water coolant. Then the exposed superficial dentin surface was polished by silicon carbide paper (600 grit) under water coolant to standardize the smear layer. The teeth were rinsed with distilled water to remove any debris. The teeth were then mounted in acrylic resin (2×3×5cm) and were randomly divided into six groups (n=10). The specimens’ preparation was based on the type of the employed adhesive systems and the tested temperatures as follows:

Group 1: Adper single Bond at 4˚C
Group 2: Adper single Bond at 25˚C
Group 3: Adper single Bond at 40˚C
Group 4: Clearfil SE Bond at 4˚C
Group 5: Clearfil SE Bond at 25˚C
Group 6: Clearfil SE Bond at 40˚C

For providing the adhesive at different temperatures (4˚C, 25˚C and 40˚C), the adhesive systems were put in refrigerator at 4˚C, and in water bath (Teledyne Hanau; Buffalo, NY, USA) to increase the adhesive temperature (40^°^C). The control groups were kept at room temperature (25^°^C). The adhesive systems were stored in the aforementioned temperatures for 30 minutes.

The adhesive systems were applied following the manufacturer’s instructions (Table 1).

**Table 1:** Adhesive systems ,Composition and Mode of Application

**Adhesive Systems**	**Composition**	**Application Mode**
Adper Single Bond 3M ESPE , USA	Adhesive: Bis-GMA; HEMA; Dimethacrylatas; Polyalkanoic acid copolymer; initiators; water; and ethanol.	Acid etch(15 s); rinse(15 s); air-dry (30 s); dentin rewetted with water (1.5 µl) (60 s); one coat of adhesive; air-dry (10s at 20cm); light cure (10s)
Clearfil SE Bond Kuraray Medical Inc, Japan	Primer: MDP; HEMA; Hydrophilic Dimethacrylate; Camphorquinone; water. Adhesive: MDP; HEMA; Bis-GMA; Hydrophobic Dimethacrylate; N, N diethanol p-toluidine; Camphorquinone bond; Silanated colloidal silica.	air-dry the dentin surface; two coats of primer with slight agitation (20s); air-dry (20s at 20cm); one coat of the adhesive with slight agitation (20s); light cure (10s)

**Figure 1:**
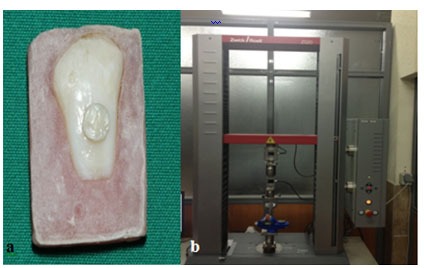
a: Composite filling on dentinal surface  b: Instron universal testing machine

The specimens in groups 1, 2 and 3 received etch-and-rinse adhesive system and were conditioned with 37% phosphoric acid (Dental Conditioning Gel; Dentsply Gaulk) for 15 seconds. The acid was then rinsed with distilled water for 15 seconds and then air-dried. The dentin was then rewetted with water for 60 seconds and received one coat of adhesive and air-dried for 10 seconds and light-cured (Optilux 501; Sybron Kerr, Danbury, CT. USA) for 10 seconds (600 ^mW^ ⁄ _Cm^2^_), constantly. In groups 4, 5 and 6, the adhesive systems were applied at different temperatures (4˚C, 25˚C and 40˚C). Based on the manufacturer’s instruction (Table 1), the dentin surface was air-dried; two coats of primer with slight agitation were applied for 20 seconds, again air-dried (20 seconds at 20 cm). Afterward, one coat of adhesive was applied with slight agitation for 20 seconds and finally light cured for 10 seconds. Subsequent to the application of adhesive on the dentin surface, a resin composite block (Z350; A2, 3M ESPE, St. Paul, MN, USA) was built up over the bonded dentin with the aid of a Teflon mold (2mm height×5mm diameter) at room temperature, followed by light-curing for 40 seconds in vertical position on composite surface (600 ^mW^ ⁄ _Cm^2^_) (Figure 1a).

The specimens were stored in distilled water at 37^°^C for one week and then were tested in shear mode using a chisel-shaped rod attached to the universal testing machine (Zwick/Roell Z020, Germany) at a cross-head speed of 1mm/min (Figure 1b) until the bond fractured and the shear force was evaluated.

**Figure 2:**
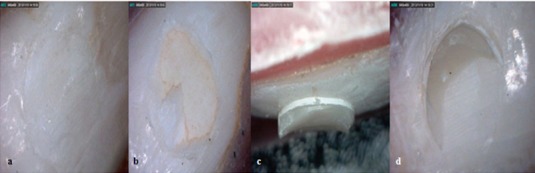
Types of fractures: a: Adhesive  b: Mix  c: Cohesive in the composite  d: Cohesive in dentin

The tested specimens were observed under a stereomicroscope (×40) (Motic K-500L; Motic Incorporation Ltd, Hong Kong) to evaluate the fracture mode (Figure 2). Table 2 represents the percentages of fracture types. [15-16] The percentages of fractures were evaluated visually and the subtypes were as following:

Type 1- Adhesive: when more than 50% of fractured surface was between the composite and dentin.
Type 2- Cohesive: when more than 50% of fractured surface was in the composite block or dentin.
Type 3- Adhesive/Cohesive: when there was the same amount of fractured surface in a sample, it was considered as mix or Adhesive/Cohesive. [15-16]

**Table 2:** Shear Bond Strength values (MPa) for the two adhesive systems at three different temperatures (mean±SD)

**Adhesive**	**Temperature**		
	**4˚C**	**25˚C**	**40˚C**
SE Bond	(10.41±1.67) Aa	(11.41±1.95) Aa	(11.21±2.16) Aa
Single Bond	(6.33±1.38) Bb	(10.31±1.38) Aa	(10.24±1.69) Aa
P*- Value	<0.001	0.162	0.282

*using student’s t-test

The mean values followed by different letters show statistical difference (One-way ANOVA/ Tukey Test)

Upper-case letters: comparison of adhesives at each temperature (column)

Lower-cases letters: comparison of temperatures for each adhesive (line)

## Results

Two-way ANOVA and subgroup analysis based on student’s t-test and One-Way ANOVA/Tukey test were employed to compare the mean of Shear Bond Strength (SBS) among the groups. Table 3 demonstrated the mean bond strength values and standard deviation of the two adhesive systems at the three tested temperature levels.

**Table 3:** Percentage of fracture types for each group

**Groups**	**Types**		
	**Type 1 (%)**	**Type 2 (%)**	**Type 3 (%)**
Group 1	60	10	30
Group 2	20	60	20
Group 3	30	60	10
Group 4	50	20	30
Group 5	40	30	30
Group 6	50	30	20

Fracture types: 1: Adhesive, 2: Cohesive,: Mixed or Adhesive/ Cohesive

**Figure 3:**
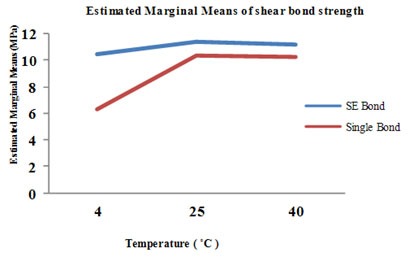
The relationship between temperature and adhesive systems

Two-way ANOVA showed that the interaction between temperature and materials was significant (*p*= 0.009). One-way ANOVA was used for each material at the three temperature levels. The single bond adhesive system at 25˚C and 40˚C were found to have represented a statistical difference in comparison to single bond at 4˚C (*p*< 0.0001); however, there was no significant difference between the single bond at 25˚C and 40˚C and SE Bond at the three different temperature levels (α=0.05) (Figure 3).

The results of fracture strength were expressed in MPa and data were analyzed before being submitted for statistical analysis‌ (One-way and Two-way ANOVA and Tukey test) (*p*< 0.05).

## Discussion

Most of the studies investigated the temperature of adhesive systems concerning post restorative procedure conditions and there have been few studies conducted on the pre-heating effect of adhesive systems. [8, 11] Therefore, this study has investigated the effect of pre-heating on shear bond strength of adhesive systems.

Substitute substrate of bovine teeth, which has already been reported to be capable of providing similar bond strength to that of human dentin, was used in this study. [17-18]

Spohr *et al.* studied the tensile bond strength of three adhesive systems to dentin after being stored at room temperature and in refrigerator for 6 months before being used; [19] one of which was Adper single bond that revealed no significant difference in the two conditions. As with refrigeration, evaporation of the solvents could not be completed, because little or no significant change was observed in the bond strength. They reported refrigeration had not any adverse effect on the bond strength. [19]

Loguercio *et al.* evaluated the adhesive temperature on bond strength efficiency and noted that increasing the temperature (37˚C or 50˚C) of the single bond adhesive system can enhance the resin-dentin bond strength. [11] However, the results of the present study indicate that higher temperatures of single bond (25˚C or 40˚C) rather than the lower temperature (4˚C) can increase the bond strength to dentin. It may result in increasing the velocity of penetration and higher evaporation of monomer contents.

It has been reported by a number of studies that cold temperature can reduce the quality of bonding; [10, 20] however, other studies demonstrated opposite results. [19-21] The composition of adhesive systems can explain these different findings. For example, those studies that have claimed similar bonding quality for refrigerated adhesives and those stored at room condition, have evaluated etch-and-rinse systems such as single bond. [19, 21] At low-temperature conditions (5˚C) the viscosity of adhesive systems elevates significantly. [9, 21-22] It has been demonstrated that the elevated viscosity of an adhesive could interfere with the substrate wetting. [23] This theoretically affects the penetration of solvated comonomers into the acid-etched demineralized dentin matrix. Another consequent of applying the adhesive at low temperature is that this adhesive layer tends to be thicker and more variable. [11] In line with previous investigations, the present study found that lower temperature (4˚C) in comparison to higher temperature (25˚C or 40˚C), decreases etch-and-rinse adhesive system bond strength.

Donmez *et al.* investigated the effect of storage temperature on bond strength of a self-etch adhesive system to pulp chamber dentin and noticed that storing at 4˚C or 23˚C (room temperature) did not affect the bond strength, while storing at 40˚C incubator for one year significantly decreased the bond strength. [24] However, the present study ignored the factor of aging and investigated the effect of temperature before performance of single bond and SE Bond adhesive system.

Sunfeld *et al.* showed that the temperature of SE Bond adhesive system affected the quality of bond strength; good clinical results were obtained at 4˚C, whereas the quality of bond decreased at 40˚C. [20] They attributed it to the fact that the bonding pattern of self-etching adhesives (such as SE Bond) depends on their ability of incorporating the smear layer. Higher temperatures can decrease the adhesive quality of SE Bond, impair the dissolution of smear layer and demineralize the dentin. [10]

Furthermore, it has been reported that low temperatures can reduce the solvents evaporation. [25] In other words, presence of a high level of water and residual solvent in the adhesive layer can decrease the bond strength. [10, 25] Single bond in the current study contained ethanol and water, and SE Bond had an amount of water and hydrophilic dimethacrylate that might have remained and caused reduction in bond strength. It seems that ethanol in association with water evaporates faster than water solely.

Although the performance of both products at cold temperature (4˚C) revealed statistical difference in the efficiency of bond strength to dentin surface, this can be related to the fact that SE primers contain low viscosity monomers and high hydrophilic concentration such as SE Bond adhesive system, compared with other adhesive systems. [21]

One of the important factors that may also have a considerable effect on the physical properties of a self-etch adhesive system is the degree of conversion. [26] The degree of conversion of monomers can be influenced by polymerization temperature and subsequently change their properties. [10, 19]

Warming increases the radical mobility, and lead to achieving additional polymerization, which ensures good results for low viscosity systems such as SE Bond. [10, 19] However, in the present study, the three different temperatures of SE bond adhesive system represented no significant difference; that might be the result of reaching the room temperature during applying the adhesive systems.

Comparing Single Bond and SE Bond adhesive systems at each temperature, no statistical difference was found in their bond strength regarding application of these two adhesive systems at room temperature. This was also reported by other researchers who recorded similar results for self-etching and etch-and-rinse adhesives, when used on dental substrate. [27-28] The present study also demonstrated no statistical difference between the two materials at 40˚C.

It is worth mentioning that comparing the findings obtained in various studies is difficult because a series of factors may be responsible for different findings. For example, the dentin substrate and using products of the same manufacturer and the operator’s effect show the need for standardizing the methodology so that the obtained results can be compared more efficiently. [29-30]

The present study focused on the temperature of the adhesive systems before using and the temperature of composite resin was the same in all groups and was the room temperature (25˚C). Therefore, it is suggested that the temperature of both adhesive system and composite resin should be evaluated before application and the result must be compared with that of the present study. 

Also mode of fracture must be considered since it can be related to bonding ability of adhesive systems. [10] The current study showed that single bond adhesive system at lower temperature represents more incidence of adhesive fractures, while at warmer temperature shows more cohesive fracture; indicating higher bond strength at warmer temperature rather than lower temperature. On the other hand, some studies denied any direct relationship between the bond strength values and mode of fracture. [15] Hence, random incidence of each fracture mode was recorded for SE Bond adhesive system and revealed no difference between the three temperatures of SE Bond.

## Conclusion

Application of single bond adhesive system at 25˚C or 40˚C can improve the quality of resin bonding to dentin in comparison to that at 4˚C at refrigerator storage. Therefore, storing this adhesive out of refrigerator could yield better results in the clinical performance of restorative procedures. Nevertheless, SE Bond adhesive system demonstrated no sensitivity to the environmental temperature, and this study found that the storage condition before using SE Bond creates no difference. 
